# 2,7-Bis(2-nitro­phen­yl)-9-octyl-9*H*-carbazole

**DOI:** 10.1107/S1600536812012780

**Published:** 2012-03-31

**Authors:** Norma Wrobel, Dieter Schollmeyer, Heiner Detert

**Affiliations:** a University Mainz, Duesbergweg 10-14, 55099 Mainz, Germany

## Abstract

The title compound, C_32_H_31_N_3_O_4_, was obtained in a Suzuki coupling of carbazole diboronic acid and bromo­nitro­benzene. In the crystal, the mol­ecule adopts a non-symmetric conformation. The carbazole ring system is approximately planar [maximum deviation from the least-squares plane = 0.039 (2) Å]. The planes of the carbazole unit and the benzene rings subtend dihedral angles of 48.42 (7) and 41.81 (6)°. The dihedral angles between the planes of the nitro­phenyl rings and the nitro groups are 44.34 (19) and 61.64 (15)°. The crystal is built from two strands of parallel mol­ecules with inter­digitated octyl chains. These strands are symmetry related by a twofold screw axis.

## Related literature

For Suzuki cross-couplings, see: Miyaura & Suzuki (1995 ▶). For the Cadogan reaction, see: Cadogan (1962 ▶). For indolocarbazoles, see: Nemkovich *et al.* (2009 ▶). For heteroanalogous carbazoles, see: Dassonneville *et al.* (2011 ▶); Letessier & Detert (2012 ▶). For the structures of aryl-substituted carbazoles and substituted *p*-terphenyls, see: Letessier *et al.* (2011 ▶); Jones *et al.* (2005 ▶); Moschel *et al.* (2011 ▶); Wrobel *et al.* (2012 ▶).
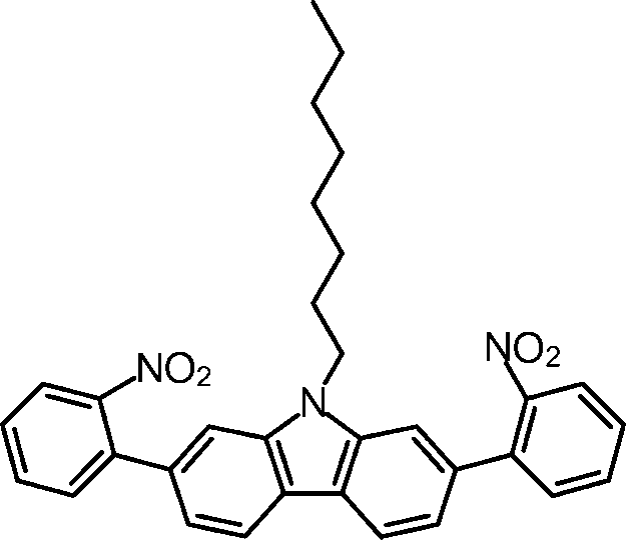


## Experimental

### 

#### Crystal data

C_32_H_31_N_3_O_4_
*M* *_r_* = 521.60
Monoclinic, 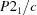

*a* = 8.722 (2) Å
*b* = 7.987 (2) Å
*c* = 39.508 (11) Å
β = 95.044 (6)°
*V* = 2741.5 (13) Å^3^
*Z* = 4
Mo *K*α radiation
μ = 0.08 mm^−1^
*T* = 173 K
0.50 × 0.04 × 0.04 mm

#### Data collection

Bruker SMART APEXII diffractometer
15053 measured reflections
6525 independent reflections
2814 reflections with *I* > 2σ(*I*)
*R*_int_ = 0.099

#### Refinement

*R*[*F* ^2^ > 2σ(*F* ^2^)] = 0.062
*wR*(*F* ^2^) = 0.137
*S* = 0.93
6525 reflections
353 parameters
H-atom parameters constrained
Δρ_max_ = 0.22 e Å^−3^
Δρ_min_ = −0.22 e Å^−3^

### 

Data collection: *APEX2* (Bruker, 2004 ▶); cell refinement: *SAINT* (Bruker, 2004 ▶); data reduction: *SAINT*; program(s) used to solve structure: *SIR97* (Altomare *et al.*, 1999 ▶); program(s) used to refine structure: *SHELXL97* (Sheldrick, 2008 ▶); molecular graphics: *PLATON* (Spek, 2009 ▶); software used to prepare material for publication: *PLATON*.

## Supplementary Material

Crystal structure: contains datablock(s) I, global. DOI: 10.1107/S1600536812012780/rz2725sup1.cif

Structure factors: contains datablock(s) I. DOI: 10.1107/S1600536812012780/rz2725Isup2.hkl

Supplementary material file. DOI: 10.1107/S1600536812012780/rz2725Isup3.cml

Additional supplementary materials:  crystallographic information; 3D view; checkCIF report

## Figures and Tables

**Fig. 1.**
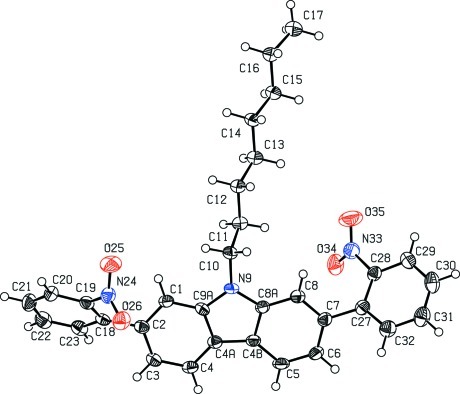
The molecular structure of the title compound. Displacement ellipsoids are drawn at the 50% probability level.

**Fig. 2.**
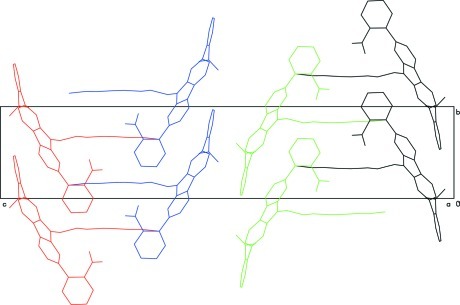
A packing section of the monoclinic crystal down the a axis. Molecules shown in equal colors are shifted by y/b = 1 along the b axis. Red/blue (green/black) molecules are related by the twofold screw axis.

## supplementary crystallographic information

### Comment

The title compound was prepared as an intermediate in a larger project on carbazoles and heteroanalogous carbazoles, see Dassonneville *et al.* (2011), Letessier *et al.* (2011), Letessier & Detert (2012). Indolocarbazoles (Nemkovich *et al.* 2009) are prepared by Cadogan reaction (Cadogan, 1962).

The molecule adopts a non-symmetric conformation with a nearly planar carbazole unit (maximum deviation from the least-squares plane = 0.039 (2) Å at C7). Attached to N9 is the octyl chain in an an *all-trans* conformation. The planes of the carbazole unit and the benzene rings subtend dihedral angles of 48.42 (7)° (ring C1–C9a and ring C18–C23) and 41.81 (6)° (ring C4b–C8a and ring C27–C32). Dihedral angles between the planes of the benzene rings and the nitro groups are 44.34 (19)° and 61.64 (35)°. Whereas the dihedral angles between the aromatic rings are comparable to those found in a *o*-nitrobiaryl with an additional *o*-substituent (Wrobel *et al.*., 2012), the dihedral angles between the planes of the niro groups and the adjacent benzene ring are even larger. Both nitro goups are oriented toward the N9 nitrogen atom of the carbazole. Two strands of parallel molecules with interdigitated octyl chains, symmetry-related by a twofold screw axis, build the crystal.

### Experimental

*N*-Octylcarbazol-2,7-diboronic acid (1 g, 1.88 mmol) and 2-bromonitrobenzene (0.75 g, 3.71 mmol) were dissolved in toluene (4.5 ml). A solution of K_2_CO_3_ (2*M*, 3 ml) was added and the mixture was stirred for 30 min. Palladium acetate (34 mg, 0.15 mmol) and triphenylphosphine (159 mg, 0.606 mmol) were added to the stirred solution. After refluxing for 48 h, the mixture was cooled, diluted with water (15 ml) and extracted with dichloromethane (3 × 25 ml). The pooled extracts were washed with water, brine, and dried (MgSO_4_). The residue was recrystallized from methanol to give the analytically pure compound. Greenish needles-like crystals suitable for X-rax analysis were grown by slow evaporation of a chloroform / methanol (1:1 *v*/*v*) solution. M. p. = 411-412 K.

### Refinement

H atoms were placed at calculated positions with C—H = 0.95 Å (aromatic) or 0.98–0.99 Å (*sp*^3^ C-atom). All H atoms were refined in the riding-model approximation with isotropic displacement parameters (set at 1.2–1.5 times of the *U*_eq_ of the parent atom).

### Crystal data

**Table:**

C_32_H_31_N_3_O_4_	*F*(000) = 1104
*M**_r_* = 521.60	*D*_x_ = 1.264 Mg m^−^^3^
Monoclinic, *P*2_1_/*c*	Melting point: 411 K
Hall symbol: -P 2ybc	Mo *K*α radiation, λ = 0.71073 Å
*a* = 8.722 (2) Å	Cell parameters from 951 reflections
*b* = 7.987 (2) Å	θ = 2.3–20.4°
*c* = 39.508 (11) Å	µ = 0.08 mm^−^^1^
β = 95.044 (6)°	*T* = 173 K
*V* = 2741.5 (13) Å^3^	Needle, green
*Z* = 4	0.50 × 0.04 × 0.04 mm

### Data collection

**Table:**

Bruker SMART APEXII diffractometer	2814 reflections with *I* > 2σ(*I*)
Radiation source: sealed Tube	*R*_int_ = 0.099
Graphite monochromator	θ_max_ = 28.0°, θ_min_ = 2.1°
CCD scan	*h* = −11→11
15053 measured reflections	*k* = −9→10
6525 independent reflections	*l* = −51→52

### Refinement

**Table:**

Refinement on *F*^2^	Primary atom site location: structure-invariant direct methods
Least-squares matrix: full	Secondary atom site location: difference Fourier map
*R*[*F*^2^ > 2σ(*F*^2^)] = 0.062	Hydrogen site location: inferred from neighbouring sites
*wR*(*F*^2^) = 0.137	H-atom parameters constrained
*S* = 0.93	*w* = 1/[σ^2^(*F*_o_^2^) + (0.0415*P*)^2^] where *P* = (*F*_o_^2^ + 2*F*_c_^2^)/3
6525 reflections	(Δ/σ)_max_ < 0.001
353 parameters	Δρ_max_ = 0.22 e Å^−^^3^
0 restraints	Δρ_min_ = −0.22 e Å^−^^3^

### Special details

**Table:**

Experimental. ^1^H-NMR (400 MHz, CDCl_3_): δ = 0.81 (m, 3 H, CH_3_), 1.20 - 1.40 (m, 10 H, CH_2_), 1.91 (qui, 2 H, β-CH_2_), 4.28 (t, 2 H, N—CH_2_), 7.18 (dd, J = 8.1 Hz, J= 1.2 Hz, 2 H), 7.34 (s, 2 H, 1-H, 8-H, carbazol), 7.49 (ddd, 2 H, 4-H phenyl), 7.56 - 7.68 (m, 4 H), 7.86 (d, 2 H, J = 7.5 Hz), 8.13 (d, 2 H, J = 8 Hz)^13^C-NMR (75 MHz, CDCl_3_): δ = 14.1, 22.6, 27.3, 28.9, 29.1, 29.3, 31.8, 43.3, 108.3, 119.2, 120.8, 122.4, 124.0, 128.0, 132.1, 132.3, 135.1, 137.0, 141.0, 149.8.ESI-MS: (*M*+H^+^): m/*z* = 522
Geometry. All e.s.d.'s (except the e.s.d. in the dihedral angle between two l.s. planes) are estimated using the full covariance matrix. The cell e.s.d.'s are taken into account individually in the estimation of e.s.d.'s in distances, angles and torsion angles; correlations between e.s.d.'s in cell parameters are only used when they are defined by crystal symmetry. An approximate (isotropic) treatment of cell e.s.d.'s is used for estimating e.s.d.'s involving l.s. planes.
Refinement. Refinement of *F*^2^ against ALL reflections. The weighted *R*-factor *wR* and goodness of fit *S* are based on *F*^2^, conventional *R*-factors *R* are based on *F*, with *F* set to zero for negative *F*^2^. The threshold expression of *F*^2^ > σ(*F*^2^) is used only for calculating *R*-factors(gt) *etc*. and is not relevant to the choice of reflections for refinement. *R*-factors based on *F*^2^ are statistically about twice as large as those based on *F*, and *R*- factors based on ALL data will be even larger.

### Fractional atomic coordinates and isotropic or equivalent isotropic displacement parameters (Å^2^)

**Table:**

	*x*	*y*	*z*	*U*_iso_*/*U*_eq_	
C1	0.1501 (3)	0.0908 (3)	0.07930 (6)	0.0281 (6)	
H1	0.2497	0.0686	0.0901	0.034*	
C2	0.0823 (3)	−0.0170 (3)	0.05459 (6)	0.0281 (6)	
C3	−0.0668 (3)	0.0158 (3)	0.03934 (6)	0.0294 (7)	
H3	−0.1128	−0.0593	0.0228	0.035*	
C4	−0.1471 (3)	0.1566 (3)	0.04823 (6)	0.0295 (6)	
H4	−0.2474	0.1779	0.0377	0.035*	
C4A	−0.0806 (3)	0.2665 (3)	0.07251 (6)	0.0268 (6)	
C4B	−0.1301 (3)	0.4184 (3)	0.08774 (6)	0.0271 (6)	
C5	−0.2635 (3)	0.5156 (4)	0.08451 (7)	0.0340 (7)	
H5	−0.3474	0.4841	0.0689	0.041*	
C6	−0.2731 (3)	0.6580 (3)	0.10414 (7)	0.0350 (7)	
H6	−0.3635	0.7248	0.1015	0.042*	
C7	−0.1520 (3)	0.7057 (3)	0.12791 (6)	0.0302 (7)	
C8	−0.0166 (3)	0.6129 (3)	0.13098 (6)	0.0303 (7)	
H8	0.0681	0.6467	0.1462	0.036*	
C8A	−0.0080 (3)	0.4701 (3)	0.11135 (6)	0.0272 (6)	
N9	0.1124 (2)	0.3575 (3)	0.11075 (5)	0.0296 (6)	
C9A	0.0684 (3)	0.2310 (3)	0.08774 (6)	0.0265 (6)	
C10	0.2587 (3)	0.3693 (3)	0.13157 (6)	0.0323 (7)	
H10A	0.2981	0.4852	0.1305	0.039*	
H10B	0.3344	0.2941	0.1221	0.039*	
C11	0.2451 (3)	0.3226 (4)	0.16865 (6)	0.0353 (7)	
H11A	0.1706	0.3987	0.1783	0.042*	
H11B	0.2049	0.2071	0.1698	0.042*	
C12	0.3993 (3)	0.3338 (4)	0.18988 (7)	0.0351 (7)	
H12A	0.4665	0.2415	0.1834	0.042*	
H12B	0.4498	0.4408	0.1849	0.042*	
C13	0.3825 (3)	0.3236 (4)	0.22768 (7)	0.0361 (7)	
H13A	0.3269	0.2191	0.2323	0.043*	
H13B	0.3183	0.4187	0.2341	0.043*	
C14	0.5335 (3)	0.3266 (4)	0.25009 (6)	0.0346 (7)	
H14A	0.5926	0.2240	0.2458	0.042*	
H14B	0.5950	0.4240	0.2437	0.042*	
C15	0.5117 (3)	0.3369 (4)	0.28762 (6)	0.0358 (7)	
H15A	0.4468	0.2416	0.2936	0.043*	
H15B	0.4549	0.4411	0.2918	0.043*	
C16	0.6591 (3)	0.3349 (4)	0.31096 (7)	0.0393 (8)	
H16A	0.7267	0.4268	0.3044	0.047*	
H16B	0.7135	0.2279	0.3079	0.047*	
C17	0.6310 (4)	0.3547 (4)	0.34818 (7)	0.0545 (9)	
H17A	0.5803	0.4622	0.3515	0.082*	
H17B	0.7296	0.3512	0.3621	0.082*	
H17C	0.5650	0.2634	0.3549	0.082*	
C18	0.1673 (3)	−0.1675 (3)	0.04443 (6)	0.0258 (6)	
C19	0.3244 (3)	−0.1658 (3)	0.03964 (6)	0.0278 (6)	
C20	0.4070 (3)	−0.3068 (3)	0.03240 (7)	0.0331 (7)	
H20	0.5144	−0.2999	0.0301	0.040*	
C21	0.3302 (4)	−0.4589 (4)	0.02854 (7)	0.0403 (8)	
H21	0.3847	−0.5576	0.0235	0.048*	
C22	0.1740 (4)	−0.4661 (4)	0.03203 (7)	0.0397 (8)	
H22	0.1205	−0.5693	0.0289	0.048*	
C23	0.0956 (3)	−0.3233 (3)	0.04001 (6)	0.0347 (7)	
H23	−0.0114	−0.3314	0.0426	0.042*	
N24	0.4094 (3)	−0.0065 (3)	0.04073 (6)	0.0338 (6)	
O25	0.5191 (2)	0.0084 (3)	0.06205 (6)	0.0545 (6)	
O26	0.3683 (2)	0.1017 (2)	0.01991 (5)	0.0440 (6)	
C27	−0.1633 (3)	0.8555 (3)	0.14989 (7)	0.0304 (7)	
C28	−0.1202 (3)	0.8539 (3)	0.18486 (6)	0.0296 (7)	
C29	−0.1115 (3)	0.9962 (4)	0.20483 (7)	0.0383 (7)	
H29	−0.0766	0.9900	0.2283	0.046*	
C30	−0.1546 (3)	1.1479 (4)	0.19000 (8)	0.0428 (8)	
H30	−0.1502	1.2473	0.2033	0.051*	
C31	−0.2040 (4)	1.1544 (4)	0.15588 (8)	0.0440 (8)	
H31	−0.2362	1.2581	0.1459	0.053*	
C32	−0.2070 (3)	1.0114 (3)	0.13615 (7)	0.0389 (7)	
H32	−0.2398	1.0193	0.1126	0.047*	
N33	−0.0912 (3)	0.6934 (3)	0.20279 (6)	0.0417 (7)	
O34	−0.1798 (3)	0.5768 (2)	0.19591 (5)	0.0500 (6)	
O35	0.0185 (3)	0.6880 (3)	0.22437 (6)	0.0664 (7)	

### Atomic displacement parameters (Å^2^)

**Table:**

	*U*^11^	*U*^22^	*U*^33^	*U*^12^	*U*^13^	*U*^23^
C1	0.0242 (16)	0.0352 (16)	0.0244 (14)	0.0019 (13)	−0.0007 (12)	0.0019 (13)
C2	0.0278 (17)	0.0343 (16)	0.0222 (13)	−0.0012 (13)	0.0027 (12)	0.0011 (13)
C3	0.0278 (17)	0.0376 (17)	0.0226 (13)	−0.0033 (14)	0.0020 (11)	−0.0034 (13)
C4	0.0222 (16)	0.0418 (17)	0.0242 (13)	0.0014 (14)	0.0004 (11)	0.0039 (14)
C4A	0.0253 (17)	0.0349 (16)	0.0208 (13)	0.0013 (13)	0.0044 (11)	0.0024 (13)
C4B	0.0257 (17)	0.0352 (16)	0.0198 (13)	0.0039 (13)	−0.0004 (11)	0.0024 (13)
C5	0.0271 (17)	0.0465 (18)	0.0268 (14)	0.0034 (14)	−0.0074 (12)	0.0002 (14)
C6	0.0317 (18)	0.0416 (18)	0.0312 (15)	0.0136 (14)	−0.0009 (13)	0.0010 (15)
C7	0.0340 (18)	0.0303 (16)	0.0264 (14)	0.0052 (13)	0.0030 (13)	0.0034 (13)
C8	0.0303 (18)	0.0339 (17)	0.0263 (14)	0.0030 (13)	−0.0008 (12)	0.0007 (13)
C8A	0.0250 (17)	0.0322 (16)	0.0241 (13)	0.0036 (13)	0.0004 (12)	0.0016 (13)
N9	0.0249 (14)	0.0373 (14)	0.0250 (11)	0.0035 (11)	−0.0076 (10)	−0.0025 (11)
C9A	0.0284 (17)	0.0319 (16)	0.0192 (12)	−0.0024 (13)	0.0017 (11)	−0.0025 (12)
C10	0.0274 (17)	0.0378 (17)	0.0303 (15)	0.0021 (13)	−0.0051 (12)	−0.0058 (14)
C11	0.0356 (18)	0.0381 (17)	0.0310 (15)	0.0054 (14)	−0.0045 (13)	−0.0007 (14)
C12	0.0349 (18)	0.0360 (17)	0.0326 (15)	0.0034 (14)	−0.0074 (13)	−0.0042 (14)
C13	0.0346 (18)	0.0395 (17)	0.0327 (15)	0.0013 (14)	−0.0052 (13)	−0.0023 (14)
C14	0.0384 (19)	0.0341 (17)	0.0294 (15)	−0.0002 (14)	−0.0072 (13)	−0.0036 (13)
C15	0.0397 (19)	0.0371 (17)	0.0294 (15)	0.0009 (14)	−0.0045 (13)	−0.0031 (14)
C16	0.0403 (19)	0.0414 (18)	0.0345 (16)	0.0066 (15)	−0.0067 (14)	−0.0060 (15)
C17	0.070 (3)	0.054 (2)	0.0366 (17)	0.0176 (18)	−0.0127 (16)	−0.0055 (17)
C18	0.0278 (17)	0.0325 (16)	0.0169 (12)	−0.0024 (13)	0.0003 (11)	−0.0004 (12)
C19	0.0345 (18)	0.0248 (15)	0.0234 (13)	−0.0033 (13)	−0.0012 (12)	0.0013 (12)
C20	0.0331 (18)	0.0313 (17)	0.0349 (16)	0.0053 (14)	0.0023 (13)	−0.0002 (14)
C21	0.053 (2)	0.0267 (17)	0.0412 (17)	0.0063 (15)	0.0052 (15)	0.0017 (14)
C22	0.053 (2)	0.0297 (17)	0.0364 (17)	−0.0093 (15)	0.0018 (15)	0.0011 (14)
C23	0.0385 (19)	0.0352 (18)	0.0301 (15)	−0.0070 (15)	0.0007 (13)	−0.0002 (14)
N24	0.0304 (16)	0.0332 (15)	0.0382 (14)	0.0016 (12)	0.0054 (12)	−0.0020 (13)
O25	0.0323 (14)	0.0598 (15)	0.0676 (15)	−0.0086 (11)	−0.0162 (12)	−0.0030 (13)
O26	0.0521 (15)	0.0321 (12)	0.0479 (13)	−0.0019 (10)	0.0062 (11)	0.0087 (11)
C27	0.0277 (17)	0.0316 (16)	0.0323 (15)	0.0016 (13)	0.0053 (12)	0.0025 (14)
C28	0.0337 (18)	0.0275 (15)	0.0283 (14)	0.0040 (13)	0.0064 (12)	0.0049 (13)
C29	0.0390 (19)	0.0394 (18)	0.0366 (16)	−0.0024 (15)	0.0046 (13)	−0.0079 (16)
C30	0.048 (2)	0.0298 (17)	0.053 (2)	−0.0068 (15)	0.0198 (16)	−0.0048 (16)
C31	0.051 (2)	0.0314 (18)	0.051 (2)	0.0019 (15)	0.0148 (16)	0.0074 (17)
C32	0.044 (2)	0.0357 (17)	0.0376 (16)	0.0007 (15)	0.0061 (14)	0.0064 (15)
N33	0.0542 (19)	0.0428 (17)	0.0287 (13)	0.0105 (14)	0.0078 (13)	0.0047 (13)
O34	0.0712 (17)	0.0279 (12)	0.0522 (14)	0.0002 (11)	0.0137 (12)	0.0040 (11)
O35	0.0716 (18)	0.0771 (18)	0.0473 (14)	0.0137 (14)	−0.0129 (13)	0.0204 (13)

### Geometric parameters (Å, º)

**Table:**

C1—C9A	1.384 (3)	C14—H14A	0.9900
C1—C2	1.393 (3)	C14—H14B	0.9900
C1—H1	0.9500	C15—C16	1.515 (4)
C2—C3	1.409 (3)	C15—H15A	0.9900
C2—C18	1.486 (4)	C15—H15B	0.9900
C3—C4	1.386 (4)	C16—C17	1.520 (4)
C3—H3	0.9500	C16—H16A	0.9900
C4—C4A	1.388 (3)	C16—H16B	0.9900
C4—H4	0.9500	C17—H17A	0.9800
C4A—C9A	1.413 (3)	C17—H17B	0.9800
C4A—C4B	1.438 (3)	C17—H17C	0.9800
C4B—C5	1.395 (4)	C18—C23	1.397 (3)
C4B—C8A	1.414 (3)	C18—C19	1.399 (4)
C5—C6	1.383 (4)	C19—C20	1.381 (3)
C5—H5	0.9500	C19—N24	1.471 (3)
C6—C7	1.403 (4)	C20—C21	1.390 (4)
C6—H6	0.9500	C20—H20	0.9500
C7—C8	1.390 (4)	C21—C22	1.382 (4)
C7—C27	1.487 (4)	C21—H21	0.9500
C8—C8A	1.385 (3)	C22—C23	1.380 (4)
C8—H8	0.9500	C22—H22	0.9500
C8A—N9	1.385 (3)	C23—H23	0.9500
N9—C9A	1.390 (3)	N24—O25	1.224 (3)
N9—C10	1.459 (3)	N24—O26	1.225 (3)
C10—C11	1.526 (4)	C27—C32	1.398 (3)
C10—H10A	0.9900	C27—C28	1.400 (4)
C10—H10B	0.9900	C28—C29	1.382 (4)
C11—C12	1.524 (4)	C28—N33	1.475 (3)
C11—H11A	0.9900	C29—C30	1.383 (4)
C11—H11B	0.9900	C29—H29	0.9500
C12—C13	1.515 (4)	C30—C31	1.379 (4)
C12—H12A	0.9900	C30—H30	0.9500
C12—H12B	0.9900	C31—C32	1.381 (4)
C13—C14	1.522 (4)	C31—H31	0.9500
C13—H13A	0.9900	C32—H32	0.9500
C13—H13B	0.9900	N33—O35	1.225 (3)
C14—C15	1.514 (4)	N33—O34	1.225 (3)
			
C9A—C1—C2	118.2 (2)	C13—C14—H14A	108.9
C9A—C1—H1	120.9	C15—C14—H14B	108.9
C2—C1—H1	120.9	C13—C14—H14B	108.9
C1—C2—C3	120.3 (2)	H14A—C14—H14B	107.8
C1—C2—C18	119.8 (2)	C14—C15—C16	115.0 (2)
C3—C2—C18	119.9 (2)	C14—C15—H15A	108.5
C4—C3—C2	120.7 (2)	C16—C15—H15A	108.5
C4—C3—H3	119.7	C14—C15—H15B	108.5
C2—C3—H3	119.7	C16—C15—H15B	108.5
C3—C4—C4A	119.9 (2)	H15A—C15—H15B	107.5
C3—C4—H4	120.1	C15—C16—C17	112.8 (3)
C4A—C4—H4	120.1	C15—C16—H16A	109.0
C4—C4A—C9A	118.7 (2)	C17—C16—H16A	109.0
C4—C4A—C4B	134.4 (3)	C15—C16—H16B	109.0
C9A—C4A—C4B	106.8 (2)	C17—C16—H16B	109.0
C5—C4B—C8A	118.1 (2)	H16A—C16—H16B	107.8
C5—C4B—C4A	135.2 (2)	C16—C17—H17A	109.5
C8A—C4B—C4A	106.6 (2)	C16—C17—H17B	109.5
C6—C5—C4B	119.8 (2)	H17A—C17—H17B	109.5
C6—C5—H5	120.1	C16—C17—H17C	109.5
C4B—C5—H5	120.1	H17A—C17—H17C	109.5
C5—C6—C7	121.3 (2)	H17B—C17—H17C	109.5
C5—C6—H6	119.4	C23—C18—C19	115.1 (2)
C7—C6—H6	119.4	C23—C18—C2	121.7 (2)
C8—C7—C6	119.8 (2)	C19—C18—C2	123.1 (2)
C8—C7—C27	118.5 (2)	C20—C19—C18	123.7 (2)
C6—C7—C27	121.7 (2)	C20—C19—N24	116.2 (2)
C8A—C8—C7	118.5 (2)	C18—C19—N24	120.1 (2)
C8A—C8—H8	120.7	C19—C20—C21	118.7 (3)
C7—C8—H8	120.7	C19—C20—H20	120.6
N9—C8A—C8	128.5 (2)	C21—C20—H20	120.6
N9—C8A—C4B	109.1 (2)	C22—C21—C20	119.7 (3)
C8—C8A—C4B	122.3 (2)	C22—C21—H21	120.2
C8A—N9—C9A	108.5 (2)	C20—C21—H21	120.2
C8A—N9—C10	125.1 (2)	C23—C22—C21	120.1 (3)
C9A—N9—C10	126.4 (2)	C23—C22—H22	120.0
C1—C9A—N9	128.9 (2)	C21—C22—H22	120.0
C1—C9A—C4A	122.3 (2)	C22—C23—C18	122.6 (3)
N9—C9A—C4A	108.9 (2)	C22—C23—H23	118.7
N9—C10—C11	112.8 (2)	C18—C23—H23	118.7
N9—C10—H10A	109.0	O25—N24—O26	123.9 (2)
C11—C10—H10A	109.0	O25—N24—C19	117.7 (2)
N9—C10—H10B	109.0	O26—N24—C19	118.3 (2)
C11—C10—H10B	109.0	C32—C27—C28	115.6 (3)
H10A—C10—H10B	107.8	C32—C27—C7	121.3 (2)
C12—C11—C10	112.0 (2)	C28—C27—C7	122.9 (2)
C12—C11—H11A	109.2	C29—C28—C27	123.5 (3)
C10—C11—H11A	109.2	C29—C28—N33	116.2 (2)
C12—C11—H11B	109.2	C27—C28—N33	120.1 (2)
C10—C11—H11B	109.2	C28—C29—C30	118.6 (3)
H11A—C11—H11B	107.9	C28—C29—H29	120.7
C13—C12—C11	112.5 (2)	C30—C29—H29	120.7
C13—C12—H12A	109.1	C31—C30—C29	119.8 (3)
C11—C12—H12A	109.1	C31—C30—H30	120.1
C13—C12—H12B	109.1	C29—C30—H30	120.1
C11—C12—H12B	109.1	C30—C31—C32	120.6 (3)
H12A—C12—H12B	107.8	C30—C31—H31	119.7
C12—C13—C14	114.7 (2)	C32—C31—H31	119.7
C12—C13—H13A	108.6	C31—C32—C27	121.7 (3)
C14—C13—H13A	108.6	C31—C32—H32	119.1
C12—C13—H13B	108.6	C27—C32—H32	119.1
C14—C13—H13B	108.6	O35—N33—O34	124.5 (3)
H13A—C13—H13B	107.6	O35—N33—C28	117.1 (3)
C15—C14—C13	113.2 (2)	O34—N33—C28	118.4 (2)
C15—C14—H14A	108.9		
			
C9A—C1—C2—C3	−1.3 (4)	C11—C12—C13—C14	177.5 (2)
C9A—C1—C2—C18	179.1 (2)	C12—C13—C14—C15	173.2 (2)
C1—C2—C3—C4	1.0 (4)	C13—C14—C15—C16	178.2 (2)
C18—C2—C3—C4	−179.3 (2)	C14—C15—C16—C17	177.0 (2)
C2—C3—C4—C4A	−0.3 (4)	C1—C2—C18—C23	136.3 (3)
C3—C4—C4A—C9A	−0.2 (4)	C3—C2—C18—C23	−43.3 (3)
C3—C4—C4A—C4B	−178.9 (3)	C1—C2—C18—C19	−41.6 (3)
C4—C4A—C4B—C5	0.3 (5)	C3—C2—C18—C19	138.7 (3)
C9A—C4A—C4B—C5	−178.4 (3)	C23—C18—C19—C20	−2.5 (4)
C4—C4A—C4B—C8A	179.5 (3)	C2—C18—C19—C20	175.6 (2)
C9A—C4A—C4B—C8A	0.7 (3)	C23—C18—C19—N24	174.8 (2)
C8A—C4B—C5—C6	−0.0 (4)	C2—C18—C19—N24	−7.2 (4)
C4A—C4B—C5—C6	179.1 (3)	C18—C19—C20—C21	2.1 (4)
C4B—C5—C6—C7	−1.2 (4)	N24—C19—C20—C21	−175.3 (2)
C5—C6—C7—C8	2.8 (4)	C19—C20—C21—C22	−0.1 (4)
C5—C6—C7—C27	−178.1 (2)	C20—C21—C22—C23	−1.4 (4)
C6—C7—C8—C8A	−3.0 (4)	C21—C22—C23—C18	0.9 (4)
C27—C7—C8—C8A	177.8 (2)	C19—C18—C23—C22	1.0 (4)
C7—C8—C8A—N9	−178.4 (3)	C2—C18—C23—C22	−177.1 (2)
C7—C8—C8A—C4B	1.8 (4)	C20—C19—N24—O25	−61.9 (3)
C5—C4B—C8A—N9	179.9 (2)	C18—C19—N24—O25	120.6 (3)
C4A—C4B—C8A—N9	0.5 (3)	C20—C19—N24—O26	116.7 (3)
C5—C4B—C8A—C8	−0.3 (4)	C18—C19—N24—O26	−60.8 (3)
C4A—C4B—C8A—C8	−179.6 (2)	C8—C7—C27—C32	127.9 (3)
C8—C8A—N9—C9A	178.5 (3)	C6—C7—C27—C32	−51.2 (4)
C4B—C8A—N9—C9A	−1.6 (3)	C8—C7—C27—C28	−47.1 (4)
C8—C8A—N9—C10	−0.1 (4)	C6—C7—C27—C28	133.7 (3)
C4B—C8A—N9—C10	179.8 (2)	C32—C27—C28—C29	−3.7 (4)
C2—C1—C9A—N9	−178.4 (2)	C7—C27—C28—C29	171.5 (3)
C2—C1—C9A—C4A	0.8 (4)	C32—C27—C28—N33	172.1 (2)
C8A—N9—C9A—C1	−178.7 (3)	C7—C27—C28—N33	−12.6 (4)
C10—N9—C9A—C1	−0.2 (4)	C27—C28—C29—C30	3.3 (4)
C8A—N9—C9A—C4A	2.1 (3)	N33—C28—C29—C30	−172.8 (3)
C10—N9—C9A—C4A	−179.4 (2)	C28—C29—C30—C31	−0.4 (4)
C4—C4A—C9A—C1	−0.0 (4)	C29—C30—C31—C32	−1.7 (4)
C4B—C4A—C9A—C1	179.0 (2)	C30—C31—C32—C27	1.1 (4)
C4—C4A—C9A—N9	179.3 (2)	C28—C27—C32—C31	1.5 (4)
C4B—C4A—C9A—N9	−1.7 (3)	C7—C27—C32—C31	−173.9 (3)
C8A—N9—C10—C11	74.3 (3)	C29—C28—N33—O35	−44.5 (4)
C9A—N9—C10—C11	−104.0 (3)	C27—C28—N33—O35	139.3 (3)
N9—C10—C11—C12	179.4 (2)	C29—C28—N33—O34	133.6 (3)
C10—C11—C12—C13	168.0 (2)	C27—C28—N33—O34	−42.6 (4)
